# The impact of microsphere deposition algorithm complexity on microdosimetry following Yttrium-90 radioembolization

**DOI:** 10.1088/2057-1976/ae5972

**Published:** 2026-04-13

**Authors:** E Courtney Henry, Benjamin P Lopez, S Cheenu Kappadath

**Affiliations:** University of Texas MD Anderson Cancer Center, Department of Imaging Physics, Houston, TX, United States of America

**Keywords:** yttrium-90, radioembolization, stochastic modeling, microdosimetry

## Abstract

The microsphere spatial distribution following Yttrium-90 radioembolization (^90^Y-RE) is inherently nonuniform, resulting in substantial microscopic dose heterogeneity not captured by conventional macroscopic dosimetry models. The motivation for this study was to build a robust framework to further understand the relationship between microdosimetry and macrodosimetry-based clinical outcomes. In this study, a stochastic microsphere deposition algorithm sampled histologically-derived cumulative distribution functions (CDFs) governing microsphere cluster diameter (${C}_{{dia}}$), distance between clusters (${C}_{{dist}}$), and cluster population (${C}_{{pop}}$). Six unique models were generated to examine the impact of algorithm complexity on the corresponding absorbed dose distribution, ranging from a completely uniform to fully stochastic reference model. A two-sample statistical Kolmogorov-Smirnov test compared ${C}_{{dia}}$, ${C}_{{dist}}$, and ${C}_{{pop}}$ derived separately from discrete and continuous CDFs. Microdosimetry calculations were performed by convolving a high-resolution dose-voxel kernel with each model. The mean absorbed dose ${D}_{{mean}}$ and various dose-volume metrics (${D}_{x},x=1,\,5,\,10,\,50,\,90,\,95,\,99$) were calculated and compared to the reference model to assess the impact of algorithm complexity on dose metric error (${E}_{x}$). Published median values of ${C}_{{dia}}$, ${C}_{{dist}}$, and ${C}_{{pop}}$ agreed well with simulated counterparts. There were no statistically significant differences in sampling between discrete and continuous CDFs for ${C}_{{dia}}$ ($p=0.083$), ${C}_{{dist}}$ ($p=0.104$), and ${C}_{{pop}}$ ($p=0.094$). Convolution with the ^90^Y dose-voxel kernel resulted a −0.3% deviation compared to a single compartment dose estimate. Model comparisons suggest that sampling ${C}_{{dist}}$​ is critical for accurately modeling low-dose regions (${E}_{99}=16 \% $), while sampling ${C}_{{pop}}$ is critical for resolving absorbed dose hot spots (${E}_{1}=-12 \% $). In contrast, sampling from ${C}_{{dia}}$​ had minimal impact on model accuracy. The results of this study provide the necessary framework to develop an improved understanding of the relationship between microdosimetry and macrodosimetry-based clinical outcomes following ^90^Y-RE.

## 1. Introduction

Radioembolization (RE) is a locoregional therapy for the treatment of unresectable primary and metastatic liver cancer. In RE, radioactive microspheres (approximately 30 μm diameter) are administered via the hepatic arterial blood supply where they get trapped in the tumor microvasculature to deliver a therapeutic absorbed dose directly to tumors, while minimizing damage to the surrounding healthy tissue. Two microsphere products are commercially available: yttrium-90 (^90^Y) glass microspheres (TheraSphere^®^, Boston Scientific, MA, USA) and ^90^Y resin microspheres (SIR-Spheres^®^, Sirtex Medical Ltd, Woburn, MA, USA). Differences exist between the commercial products with respect to the size, density, specific activity, and administered number of spheres [1].

Following RE, the mean absorbed dose (D_mean_) to a macroscopic volume has been shown to correlate with tumor response and normal tissue toxicity [2–5]. While D_mean_, when calculated using a single or multi-compartment dosimetry model, is an appropriate metric to describe macroscopic intra-compartment uniform dose distributions, it is incapable of reflecting the true dose inhomogeneity present at the microscopic scale [6]. This is particularly relevant for RE as microspheres deposit in clusters to form highly nonuniform spatial distributions [7]. Through the analysis of explanted tissue samples, a landmark ^90^Y-RE study quantified *in vivo* microsphere cluster parameters in the tumor periphery where the majority microspheres are found. These parameters included the cluster diameter (${C}_{{dia}}$), the minimum distance between clusters (${C}_{{dist}}$), and the microsphere population within each cluster (${C}_{{pop}}$) [7]. Median values for these parameters were determined to be 337 μm for ${C}_{{dia}}$, 470 μm for ${C}_{{dist}}$, and 5 microspheres for ${C}_{{pop}}$.

The consequences of microsphere distribution nonuniformity are evident in the dosimetry literature. A study examining tumor tissue samples following ^90^Y-RE found that D_mean_ on the tumor periphery ranged from 200 to 600 Gy, while D_mean_ at the center of the tumor was only 7 Gy [8]. A subsequent study analyzed explanted human livers following ^90^Y-RE and found the point dose varied from 100 Gy to more than 3,000 Gy across a single tumor volume [9]. Another study found D_mean_ was between 9 Gy and 246 Gy over 21 independent biopsy samples following ^90^Y-RE [10]. These results demonstrate the need to investigate microscopic characteristics that may impact tumor response beyond D_mean_ evaluated at a macroscopic scale.

More recently, the impact of microsphere concentration and specific activity on patient outcome, using single or multi-compartment dosimetry, has become a topic of growing interest in RE [11, 12]. Previous studies suggest that an increase in the microsphere concentration results in an increased biological effect [13, 14], but also exercise caution in administering excessively high concentrations to avoid healthy tissue toxicity [15]. A clinical study argued that the microsphere specific activity is the most relevant parameter to predict tumor response [16], while a subsequent editorial concluded that treatment efficacy is decreased if the microsphere concentration is too high, or if the microsphere specific activity is too low [12]. This growing body of work reflects the current uncertainty in choosing the appropriate balance of microsphere concentration and specific activity, for a given mean absorbed dose, to maximize tumor response and minimize normal tissue toxicity.

A computational study previously investigated the effect of microsphere concentration on tumor control using ^90^Y microsphere histological data and a stochastic algorithm to generate realistic microsphere distributions [14]. While the study found that changes in microsphere concentration may impact dose heterogeneity and explain differences in treatment planning strategies between SIR-spheres^®^ and TheraSphere^®^ microspheres, the potential of the algorithm for generating realistic microsphere distributions merits further investigation. The ability to successfully model microsphere deposition enables the study of previously inaccessible variables, such as microsphere concentration and specific activity, and their impact on the absorbed dose distribution and patient outcome.

A thorough quantitative evaluation of the microsphere deposition algorithm is required before it can be successfully used to investigate the optimal combination of microsphere concentration and specific activity. Furthermore, given that the algorithm relies on previously published histological data, there are limitations associated with sampling the discrete cumulative distribution functions (CDFs) describing ${C}_{{dia}}$, ${C}_{{dist}}$, and ${C}_{{pop}}$. For example, discrete probability distribution functions (PDFs) need to be manually extracted from the research article, originally published in the year 2000. The PDFs also contain noise from small sample sizes. These limitations motivate the need for fitting the discrete PDFs with analytical functions to provide a more robust sampling approach.

The objectives of this study are to (1) populate various mathematical *in vivo* ^90^Y microsphere models using a stochastic microsphere deposition algorithm based on explant data, (2) evaluate the accuracy of the models and compare sampling methodologies, (3) calculate the microscopic dose distribution through convolution of the ^90^Y activity distribution with a high-resolution dose-voxel kernel, and (4) investigate the impact of sampling the ${C}_{{dia}}$, ${C}_{{dist}}$, and ${C}_{{pop}}$ distributions (and therefore the computational complexity) on the accuracy of absorbed dose metrics. The results of this study will provide the necessary framework to develop an improved understanding of the relationship between microdosimetry and clinical outcomes following ^90^Y-RE.

## 2. Methods

### 2.1. Mathematical models

*In vivo* mathematical models with a side length of 2.2 cm and volume 10.65 cm^3^ were divided into 30 μm isotropic voxels (27,000 μm^3^) to produce a high-resolution matrix (735 × 735 × 735) using MATLAB (version 2024a, MathWorks Inc., Natick, MA, USA). Published histological data describing ${C}_{{dia}}$, ${C}_{{dist}}$, and ${C}_{{pop}}$ were converted into discrete PDFs referred to as ${{PDF}}_{d}^{{dia}}$, ${{PDF}}_{d}^{{dist}}$, and ${{PDF}}_{d}^{{pop}}$, respectively [7]. Discrete PDFs were fit with analytical functions to produce continuous analogues ${{PDF}}_{c}^{{dia}}$, ${{PDF}}_{c}^{{dist}}$, and ${{PDF}}_{c}^{{pop}}$. Based on a qualitative inspection of the discrete PDFs, a 4-parameter biexponential model (equations (1)–(2)) was chosen for ${{PDF}}_{c}^{{dia}}$ and ${{PDF}}_{c}^{{pop}}$ while a 3-parameter generalized extreme value model (equation (3)) was chosen for ${{PDF}}_{c}^{{dist}}$, although these functions may not be unique in describing the PDFs. For example, a recent study modeled ${{PDF}}_{d}^{{pop}}$ using a lognormal distribution [17].

\begin{eqnarray*}{{PDF}}_{c}^{{dia}}\left(x\right)=\alpha {e}^{\beta x}+\gamma {e}^{\delta x}\end{eqnarray*}

\begin{eqnarray*}{{PDF}}_{c}^{{pop}}\left(x\right)=a{e}^{{bx}}+c{e}^{{dx}}\end{eqnarray*}

\begin{eqnarray*}{{PDF}}_{c}^{{dist}}\left(x\right)={e}^{-{\left[1+k\left(\frac{x-\mu }{\sigma }\right)\right]}^{-\frac{1}{k}}}\end{eqnarray*}

Parameter estimates and 95% confidence intervals (CIs) for all parameters were determined through non-linear least squares fitting in MATLAB.

Discrete PDFs were integrated to generate CDFs, which were randomly sampled to populate the models [14]. Microsphere cluster coordinates were determined using a rejection sampling method. Briefly, a cluster of random ${C}_{{dia}}$ was randomly placed within the bounds of the model. Successive cluster locations were determined by randomly placing additional clusters and calculating ${C}_{{dist}}$ between all existing clusters. Clusters whose value of ${C}_{{dist}}$ deviated from ${{PDF}}_{c}^{{dist}}$ were rejected, while non-rejected clusters were randomly assigned a ${C}_{{pop}}$ value. The number of microspheres determined from ${C}_{{pop}}$ were randomly placed within the bounds of the cluster. A preclinical study demonstrated that ${C}_{{dist}}$ is inversely related to microsphere concentration [15]. We therefore allowed ${C}_{{dist}}$ to vary with microsphere concentration by sampling ${{CDF}}_{d}^{{dist}}$ to generate cluster locations for the microsphere concentration described in the original study [7], then randomly selected and populated clusters with microspheres until a desired microsphere concentration was achieved.

Using the microsphere deposition algorithm, six unique models were generated to examine the impact of algorithm complexity on the corresponding absorbed dose distribution.

1. ${M}_{{ref}}$: A reference model established by randomly sampling all discrete CDFs.
2. ${M}_{{med}}$: Assigned only median values to each cluster parameter.
  Three additional models were generated by sampling the CDF for a single cluster parameter while assigning median values for the remaining two:
3. ${M}_{{dia}}$: sampled ${{CDF}}_{d}^{{dia}}$ and used median values for ${C}_{{dist}}$ and ${C}_{{pop}}$.
4. ${M}_{{dist}}$: sampled ${{CDF}}_{d}^{{dist}}$ and used median values for ${C}_{dia}$ and ${C}_{pop}$.
5. ${M}_{pop}$: sampled $CD{F}_{d}^{pop}$ and used median values for ${C}_{dia}$ and ${C}_{dist}$.
  A final model consisted of uniformly distributed microspheres.
6. ${M}_{uni}$: no sampling required as microspheres were equally spaced throughout the model.

For each model, twelve microsphere distributions were generated with concentrations between 5,000 microspheres/mL and 60,000 microspheres/mL in increments of 5,000 microspheres/mL. The model ${M}_{ref}$ was independently generated five times to assess variability in ${C}_{dia}$, ${C}_{dist}$, and ${C}_{pop}$.

### 2.2. Microdosimetry

Microdosimetry calculations were performed by convolving a high-resolution dose-voxel kernel (DVK) with the microsphere distribution ($M{S}_{dist}$) in each model, assuming each microsphere was a 30 μm voxelized source of uniformly distributed ^90^Y. The DVK was calculated through Monte Carlo radiation transport simulations in TOPAS v3.7 [18]. In these simulations, the central voxel of a matrix matching the dimensions of the model was occupied with a uniformly distributed ^90^Y source. The source voxel was set to decay for 10 million histories while the deposited energy was scored in each voxel. Particles were tracked until they left the simulation volume or had an energy <100 eV. The voxel material designation was G4_TISSUE_SOFT_ICRP having a density of 1.03 g ml^−1^ [19]. The Geant4 standard electromagnetic physics package option 4 physics list was utilized for all simulations. To determine statistical variability, the simulation was repeated ten times for a total of 100 million histories to calculate the standard deviation within each voxel (SD_DVK_). The DVK used for microdosimetry was the average DVK ($DV{K}_{avg}$) across the ten simulations. The impact of the source voxel composition on the DVK was investigated by modeling the source voxel as polystyrene and yttrium-aluminium-silicon (YAS) glass to account for the composition of SIR-spheres and TheraSphere microspheres, respectively. Compositions for these microsphere products were derived from a previous study [20].

The absorbed dose to a voxel $D\left(x,y,z\right)$ was calculated by convolving the time-integrated activity ($\tilde{A}$) with $DV{K}_{avg}$. Since microspheres are permanent implants having no biological redistribution following their administration, $\tilde{A}$ can be expressed as the product of the ^90^Y mean lifetime ($\tau $), the microsphere’s specific activity (${A}_{MS}$), and $M{S}_{dist}$, as shown in equation (4).

\begin{eqnarray*}\begin{array}{c}\begin{array}{lll}D\left(x,y,z\right) &amp; = &amp; \tilde{A\,}\left(x^{\prime} ,y^{\prime} ,z^{\prime} \right)\,\otimes \,DVK\,\left(x,y,z\right)\\ &amp; = &amp; \tau {A}_{MS}\,\left[M{S}_{dist}\left(x^{\prime} ,y^{\prime} ,z^{\prime} \right)\,\otimes \,DV{K}_{avg}\left(x,y,z\right)\right]\\ &amp; = &amp; \tau {A}_{MS}\displaystyle \displaystyle \sum _{x^{\prime} }\displaystyle \displaystyle \sum _{y^{\prime} }\displaystyle \displaystyle \sum _{z^{\prime} }M{S}_{dist}\left(x^{\prime} ,y^{\prime} ,z^{\prime} \right)\times DV{K}_{avg}\\ &amp; &amp; \times \,\left(x-x^{\prime} ,y-y^{\prime} \,{,}z-z^{\prime} \right)\end{array}\end{array}\end{eqnarray*}

The sampling frequency of ${A}_{MS}$ varied depending on its magnitude: 1 Bq increments from 1 to 50 Bq, 10 Bq increments from 50 to 100 Bq, and 50 Bq increments from 150 to 2500 Bq. Due to the large matrix size of $M{S}_{dist}$ and $DV{K}_{avg}$, convolution calculations were executed on a computational cluster to achieve reasonable computation times. The absorbed dose matrix was contracted by 80 voxels (2.4 mm ^90^,Y beta range) from the edge of model boundary in each dimension to restrict microdosimetry analysis to regions capable of maintaining charged particle equilibrium.

The high-resolution $DV{K}_{avg}$ was validated by comparing D_mean_ calculated from a convolution between a 1 GBq ^90^Y voxel source and $DV{K}_{avg}$, to the mean dose calculated from single compartment dosimetry (${D}_{SC}$), as shown in equation (5).

\begin{eqnarray*}{D}_{SC}=\displaystyle \frac{{S}_{90Y}* A}{M}\end{eqnarray*}

The parameter $A$ is the total activity [GBq] within the mass $M$ [kg]. The ^90^Y-specific constant ${S}_{90Y}=49.38\,J/GBq$ was calculated based on the average ^90^Y beta energy (1.485 × 10^−13^ J) and decay constant $\lambda =3.006\,\times \,{10}^{-6}\,{{\mathrm{s}}}^{-1}$ [21]. $DV{K}_{avg}$ was also validated by comparing its profile to a previously published ^90^Y dose-point kernel (DPK) profile from ICRU Report 72 [22].

### 2.3. Statistical analysis

A two-sample statistical Kolmogorov-Smirnov (KS) test compared ${C}_{dia}$, ${C}_{dist}$, and ${C}_{pop}$ derived separately from discrete and continuous CDFs to determine if the analytical functions in equations (1)–(3) accurately describe the discrete PDFs. The microsphere deposition algorithm was validated in ${M}_{ref}$ through a quantitative comparison of published and simulated statistics for ${C}_{dia}$, ${C}_{dist}$, and ${C}_{pop}$ when sampled from the discrete CDFs. Statistics include the median, 25th and 75th percentiles, minimum, and maximum values. The percent difference between published and simulated statistics was also reported. Variability in ${C}_{dia}$, ${C}_{dist}$, and ${C}_{pop}$ was assessed by measuring the standard deviation in these parameters across five simulations of ${M}_{ref}$.

For each model, D_mean_, the standard deviation of the absorbed dose ($S{D}_{dose}$), coefficient of variation ($COV$), and various dose-volume metrics were calculated. These include the median tumor dose (D_50_) and surrogates for radiographic/pathologic response (D_99_, D_90_, D_95_) and for absorbed dose hot spots (D_01_, D_05_, D_10_). For each model, the percent error in dose metrics (E_mean_, E_99_, E_95_, E_90_, E_50_, E_10_, E_05_, E_01_), relative to the corresponding metric derived from ${M}_{ref}$, was also reported.

To assess the magnitude of dose metric error against the computational burden of generating each model, the computation time to generate cluster positions, populate clusters with microspheres, and perform the convolution was recorded. These values were expressed relative to the computation time for ${M}_{ref}$. Computation time was only calculated for microsphere concentrations between 5,000 and 45,000 microspheres/mL since not all models could achieve concentrations greater than 45,000 microspheres/mL due to constraints on the maximum value of ${C}_{pop}$.

## 3. Results

### 3.1. Mathematical models

**Figure 1.**
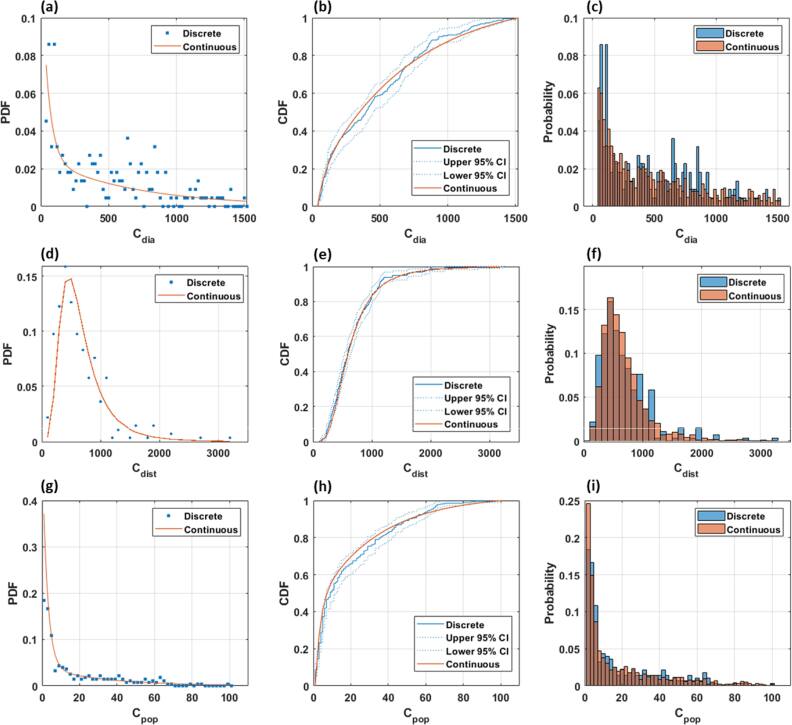
Discrete and continuous probability distribution functions for ${C}_{dia}$, ${C}_{dist}$, and ${C}_{pop}$ (a), (d), (g). Discrete (with 95% confidence intervals) and continuous cumulative distribution functions for ${C}_{dia}$, ${C}_{dist}$, and ${C}_{pop}$ (b), (e), (h). Histograms of 1,000 random samples for ${C}_{dia}$, ${C}_{dist}$, and ${C}_{pop}$ (c), (f), (i).

For each PDF in equations (1)–(3), parameter value and 95% confidence intervals are given in table 1. The results of the two-sample KS test did not show statistically significant differences in random sampling between discrete and continuous CDFs for ${C}_{dia}$ ($p=0.083$), ${C}_{dist}$ ($p=0.104$), and ${C}_{pop}$ ($p=0.094$). Furthermore, continuous CDFs are almost exclusively contained with the 95% CIs of the discrete CDFs. Continuous and discrete PDFs are shown in figures 1(a), (d), (g) for ${C}_{dia}$, ${C}_{dist}$, and ${C}_{pop}$ respectively. The corresponding CDFs are shown in figures 1(b), (e), (h), while histograms for 1,000 random samples are shown in figures 1(c), (f), (i).

**Table 1.** Parameter values and 95% confidence intervals for probability distribution functions describing cluster diameter ($PD{F}_{c}^{dia}$), distance between clusters ($PD{F}_{d}^{dist}$), and cluster population ($PD{F}_{c}^{pop}$).

Probability distribution function (PDF)	Parameter	Value	Lower 95% confidence interval	Upper 95% confidence interval
$PD{F}_{c}^{pop}$	a	$0.0389$	$0.0276$	$0.0502$
	b	$-0.0300$	$-0.0392$	$-0.0208$
	c	$0.4760$	$0.4522$	$0.4997$
	d	$-0.3706$	$-0.4058$	$-0.3355$
$PD{F}_{c}^{dia}$	$\alpha $	$0.0253$	$0.0177$	$0.0329$
	$\beta $	$-0.0015$	$-0.0020$	$-0.0010$
	$\gamma $	$0.1132$	$-0.0415$	$0.2679$
	$\delta $	$-0.0199$	$-0.0036$	$-0.0361$
$PD{F}_{d}^{dist}$	$\mu $	$445.5$	$412.5$	$478.6$
	$\sigma $	$233.9$	$207.0$	$250.7$
	$k$	$0.2118$	$0.0869$	$0.3368$

Published median values of ${C}_{dia}$, ${C}_{dist}$, and ${C}_{pop}$ are 337 μm, 470 μm, and 5 microspheres, respectively [7]. Over the five repeated simulations for ${M}_{ref}$ having a microsphere concentration of 60,000 microspheres/mL, the median values remained stable with a mean ± standard deviation of 349 ± 6, 518 ± 2, and 7 ± 1 for ${C}_{dia}$, ${C}_{dist}$, and ${C}_{pop}$, respectively. These simulated values were independent of microsphere concentration in ${M}_{ref}$. Table 2 shows additional statistical metrics for published and simulated values of ${C}_{dia}$, ${C}_{dist}$, and ${C}_{pop}$, including extrema, percentiles, and percent differences between published and simulated values.

**Table 2.** Statistics for published^7^ and simulated cluster parameters: cluster diameter ${C}_{dia}$, distance between clusters ${C}_{dist}$, and cluster population ${C}_{pop}$.

Statistic	C_dia_			C_dist_			C_pop_		
	Published [μm]	Simulated [μm]	Percent difference [%]	Published [μm]	Simulated [μm]	Percent difference [%]	Published [#]	Simulated [#]	Percent difference [%]
Median	337	349 ± 6	3.6	470	518 ± 2	10.2	5	7 ± 1	40.0
25% Percentile	101	120 ± 2	18.8	303	368 ± 2	21.5	2	3 ± 1	50.0
75% Percentile	690	713 ± 6	3.3	774	775 ± 10	0.1	14	25 ± 1	78.6
Minimum	21	20 ± 1	−4.8	89	101 ± 4	13.5	1	1 ± 0	0
Maximum	1489	1518 ± 2	1.9	3105	3205 ± 144	3.2	98	99 ± 3	1.0

### 3.2. Microdosimetry

For $DV{K}_{avg}$ validation, the absorbed dose was calculated according to equation (4) with a single 1 GBq ^90^Y source in the central voxel of a 735 × 735 × 735 matrix. The resulting ${D}_{mean}$ was compared to ${D}_{SC}$ (equation (5)) using ${K}_{90Y}=49.38\,{\mathrm{J}}\,{{\mathrm{GBq}}}^{-1}$, $M=10.65\,{\mathrm{g}}$, and $A=1\,{\mathrm{GBq}}$. The percent difference between ${D}_{mean}$ and ${D}_{SC}$ was −0.3%.

**Figure 2.**
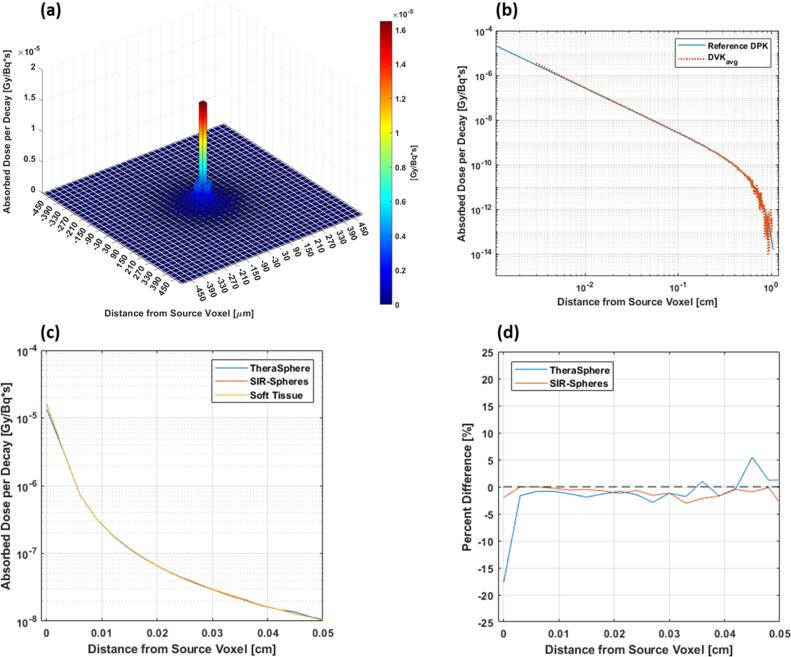
(a) Central 2D slice (area of 1 mm^2^) through the 3D dose-voxel kernel (DVK) demonstrating the dose deposition from ^90^Y decay. (b) A radial profile of the ^90^Y DVK through the central voxel compared with a ^90^Y reference kernel from ICRU Report 72. (c) Radial profiles of the ^90^Y DVK through the central voxel when that had a material composition corresponding to soft tissue, SIR-Spheres, and TheraSphere microspheres. (d) The percent difference of radial profiles in (c) relative to the profile generated with soft tissue as the composition of the central voxel.

A 2D cross-section through the central voxel of $DV{K}_{avg}$ is shown in figure 2(a) for an area of 1 mm^2^ to highlight the local dose deposition and rapid dose falloff with distance away from the source voxel. A $DV{K}_{avg}$ profile through the source voxel, averaged over the six cardinal axes, is shown in figure 2(b) on a log–log plot. In the $DV{K}_{avg}$ profile, the absorbed dose per decay in the source voxel was 1.6964 ± 0.0007 × 10^–5^. At 2.4 mm away from the source voxel (i.e., mean ^90^Y beta particle range), the absorbed dose was 3.7149 ± 0.3244 × 10^–10^. A reference ^90^Y DPK profile is shown in figure 2(b) for comparison [22]. Both $DV{K}_{avg}$ and $S{D}_{DVK}$ are provided in **Supplemental Document 1**.

$DV{K}_{avg}$ profiles from the simulations where the source voxel composition was polystyrene (SIR-Spheres), YAS glass (TheraSphere microspheres), and soft tissue are shown in figure 2(c), while the percent difference of profiles, relative to the soft tissue, is shown in figure 2(d). The percent difference in the source voxel relative to soft tissue was −17.7% and −2.0% for YAS glass and polystyrene, respectively.

**Figure 3.**
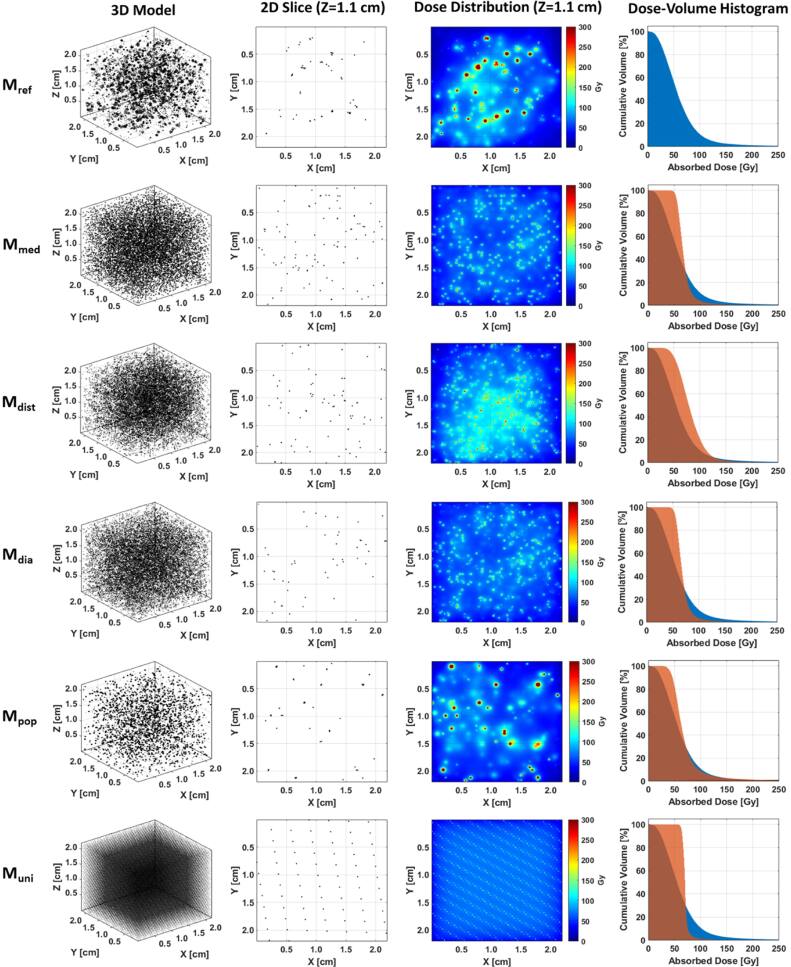
3D and 2D microsphere distributions with 5,000 microspheres/mL and 300 Bq/MS and corresponding dose distributions and dose-volume histograms. Row 1: ${M}_{ref}$. Row 2: ${M}_{med}$. Row 3: ${M}_{dist}$. Row 4: ${M}_{dia}$. Row 5: ${M}_{pop}$. Row 6: ${M}_{uni}$.

All models, corresponding dose distributions, and dose-volume histograms (DVHs) are shown in figure 3 for a microsphere concentration of 5,000 microspheres/mL and specific activity of 300 Bq/MS. Values of D_mean_, SD_dose_, COV, and E_mean_ for the models are provided in table 3. There is broad agreement in D_mean_ with values between 70.3 Gy and 83.6 Gy and a maximum E_mean_ of −15.9%. When the microsphere concentration is increased to 45,000 microspheres/mL for improved dose homogeneity, D_mean_ had a range of 115.6 Gy and a maximum E_mean_ of −15.4%, suggesting E_mean_ is independent of the microsphere concentration.

**Table 3.** Mean, standard deviation, and coefficient of variation of the absorbed dose for each model assuming a microsphere concentration of 5,000 microspheres/mL and specific activity of 300 Bq/MS.

Model	D_mean_ (Gy)	SD_dose_ (Gy)	COV	E_mean_ (%)
${M}_{ref}$	83.6	78.8	0.94	—
${M}_{med}$	70.3	29.1	0.41	−15.9
${M}_{uni}$	71.6	23.5	0.33	−14.4
${M}_{pop}$	71.7	59.6	0.83	−14.2
${M}_{dia}$	70.4	32.1	0.46	−15.8
${M}_{dist}$	83.7	37.7	0.45	0.1

The qualitative appearance of the dose distributions varies considerably. Dose distributions from ${M}_{ref}$ and ${M}_{pop}$ contain discernable absorbed dose hot spots made possible by sampling $CD{F}_{d}^{pop}$. Dose distributions from ${M}_{med}$ and ${M}_{dia}$ appear similar due to the consistent cluster spacing from the lack of sampling $CD{F}_{d}^{dist}$. Despite using median values for ${C}_{pop}$ and ${C}_{dia}$, the dose distribution of ${M}_{dist}$ still demonstrates notable dose heterogeneity from sampling $CD{F}_{d}^{dist}$. The microscopic dose inhomogeneity is further highlighted by comparing the DVHs from each model to the DVH from ${M}_{ref}$.

**Figure 4.**
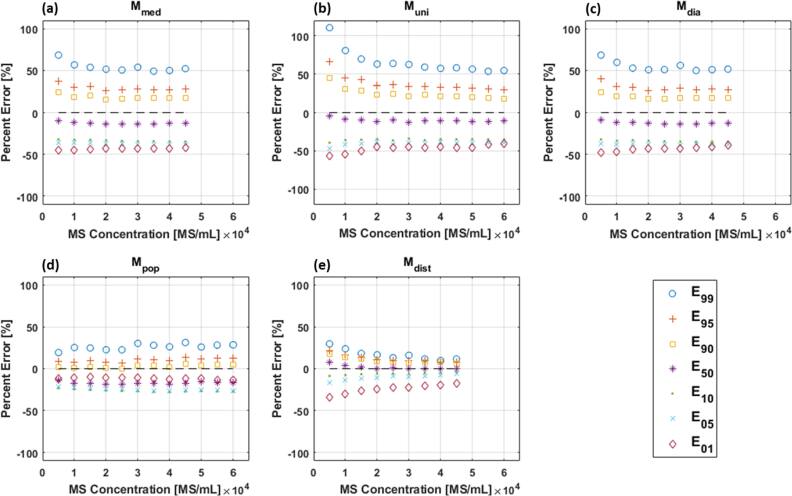
Percent error in dose-volume metrics (E_99_, E_95_, E_90_, E_50_, E_10_, E_05_, E_01_) from each model relative to the reference model ${M}_{ref}$ as a function of microsphere concentration. (a) ${M}_{med}$. (b) ${M}_{uni}$. (c) ${M}_{dia}$. (d) ${M}_{pop}$. (e) ${M}_{dist}$. The dotted line indicates perfect agreement (0% error). Models ${M}_{med}$, ${M}_{dist}$, and ${M}_{dia}$ can only accommodate microsphere concentrations up to 45,000 microspheres/mL due to constraints on the magnitude of ${C}_{pop}$.

For each model, the error in dose-volume metrics E_99_, E_95_, E_90_, E_50_, E_10_, E_05_, and E_01_ are shown in figure 4 as a function of microsphere concentration. The median and range of errors across all microsphere concentrations are presented in table 4. The data showed that ${M}_{uni}$ is the least accurate model. Error metrics E_99_ and E_01_ had median values of 61.9% and −45.4%, respectively—the highest errors across all models. The trends observed for ${M}_{uni}$ were also present in ${M}_{med}$, although the error magnitude was moderately reduced except for E_50_, which had a median value equal to −13.0%. Error metrics for ${M}_{dia}$ were similar to those derived for ${M}_{med}$. The two remaining models, ${M}_{dist}$ and ${M}_{pop}$, produced error metrics with notably reduced median errors compared to other models with most errors less than ±25%. ${M}_{pop}$ had the lowest median error for E_01_ (−11.6%) while ${M}_{dist}$ had the lowest median error for E_99_ (16.1%). Note that error metrics are independent of microsphere specific activity.

**Table 4.** The median (range) of the dose-volume metric errors across all microsphere concentrations for each model.

Model	Median (range) of percent error [%]						
	E_99_	E_95_	E_90_	E_50_	E_10_	E_05_	E_01_
M_med_	52.2 (49.2, 68.4)	28.2 (26.6, 37.4)	17.6 (16.1, 24.5)	−13.0 (−13.6, −9.9)	−34.1 (−34.8, −32.2)	−37.5 (−38.1, −36.3)	−43.0 (−45.1, −42.2)
M_dist_	16.1 (9.8, 29.8)	10.2 (8.1, 21.6)	8.0 (6.1, 17.7)	0.4 (−0.1, 8.1)	−5.8 (−8.7, −4.0)	−10.2 (−16.5, −6.2)	−22.5 (−34.5, −17.6)
M_dia_	51.6 (49.9, 68.4)	27.9 (26.6, 40.1)	17.7 (16.6, 24.5)	−13.1 (−13.8, −8.5)	−34.4 (−34.9, −32.2)	−38.0 (−38.7, −37.2)	−43.0 (−48.0, −39.3)
M_pop_	26.1 (19.3, 31.4)	10.5 (6.5, 14.0)	3.1 (−0.3, 5.7)	−17.5 (−18.4, −14.1)	−26.8 (−27.9, −23.3)	−25.1 (−26.2, −21.2)	−11.6 (−14.1, −9.9)
M_uni_	60.7 (53.5, 110.5)	34.2 (29.7, 66.5)	22.2 (18.5, 44.9)	−11.0 (−12.4, −4.4)	−35.0 (−39.4, −34.1)	−39.2 (−46.5, −38.5)	−45.7 (−56.5, −40.9)

The data shows that sampling both $CD{F}_{d}^{dist}$ and $CD{F}_{d}^{pop}$ are required for accurate modeling of low and high dose regions, respectively. Therefore, a seventh model (${M}_{dist,\,pop}$) was generated that simultaneously sampled both $CD{F}_{d}^{dist}$ and $CD{F}_{d}^{pop}$, but not $CD{F}_{d}^{dia}$. When applying the analysis used for the previous models, it was found that ${D}_{mean}$ ± $S{D}_{dose}$ was 90.5 ± 73.2 Gy for a microsphere concentration of 5,000 microspheres/mL and specific activity of 300 Bq/MS. This corresponds to a COV of 0.81 and an E_mean_ of 8.3%. The median value of the dose-volume error metrics (E_99_, E_95_, E_90_, E_50_, E_10_, E_05_, and E_01_) across all microsphere concentrations were −36.1%, −17.9%, −8.7%, 9.3%, 10.8%, 11.3%, and 19.8%, respectively. Similar to ${M}_{dist}$ and ${M}_{pop}$, the hybrid model outperformed ${M}_{uni}$, ${M}_{med}$, and ${M}_{dia}$. It did not, however, consistently outperform either ${M}_{dist}$ or ${M}_{pop}$.

**Figure 5.**
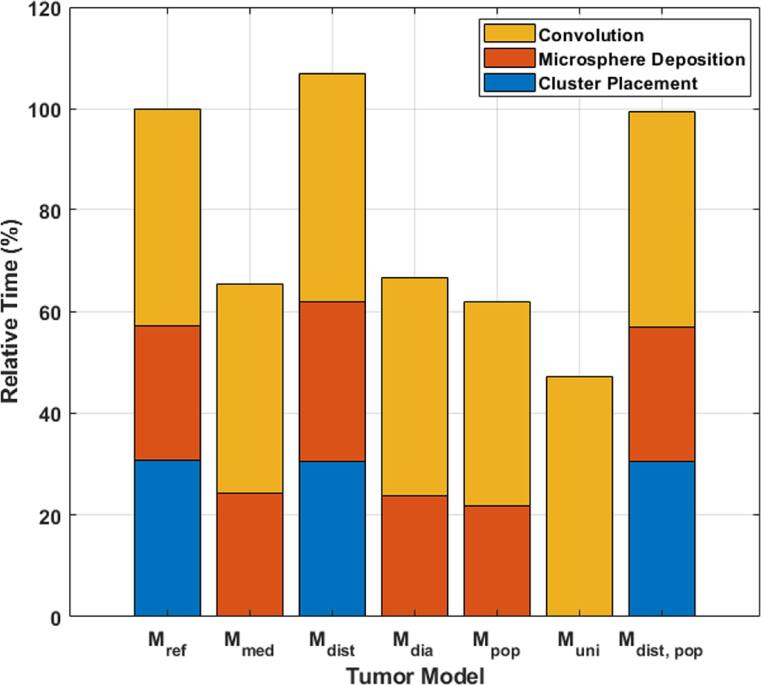
The computation time required to generate each model in terms of cluster placement, microsphere deposition within the cluster, and convolution of the dose-voxel kernel and the microsphere distribution.

Across all models, the total computation time varied between 8.3 and 18.8 h. The total computation time relative to ${M}_{ref}$ is shown in figure 5. The time taken for each step was roughly equivalent across all models with mean calculation times of 5.4, 4.5, and 7.6 h for cluster placement, microsphere deposition, and convolution, respectively. For each model, the computation time for convolution and microsphere deposition scaled linearly with the magnitude of the microsphere concentration.

Generating ${M}_{uni}$ took the least amount of time but was the most inaccurate in terms of dose metric errors. The time to generate ${M}_{med}$, ${M}_{dia}$, and ${M}_{pop}$ was roughly equivalent due to the lack of $CD{F}_{d}^{dist}$ sampling. This allowed for the cluster grid to be calculated in a negligible amount of time (<1 s), although dose metric errors for ${M}_{med}$ and ${M}_{dia}$ are sufficiently large such that the benefits of a reduced computation time must be considered. ${M}_{ref}$, ${M}_{dist}$, and ${M}_{dist,\,pop}$ had the greatest computation time due to the need for cluster placement, although sampling $CD{F}_{d}^{dist}$ was shown to be critical and producing accurate dose metrics.

## 4. Discussion

A stochastic microsphere deposition algorithm was developed using histologically derived sampling to generate accurate models for microsphere distribution. Simulated parameters for ${C}_{dia}$, ${C}_{dist}$, and ${C}_{pop}$ demonstrated strong agreement with published reference data. Results from the KS test support the use of analytical functions to fit discrete PDFs, enhancing the reproducibility and applicability of the stochastic modeling. A high-resolution ^90^Y DVK was generated for microdosimetry applications and showed a negligible deviation in the mean absorbed dose compared to a single compartment estimate. Microscopic dose distributions within the models were computed, and the influence of deposition algorithm complexity on microdosimetry was systematically assessed. Findings indicate that sampling from $CD{F}_{d}^{dist}$​ is critical for accurately modeling low-dose regions, while $CD{F}_{d}^{pop}$ is essential for resolving absorbed dose hot spots. In contrast, sampling from $CD{F}_{d}^{dia}$​ had minimal impact on overall model accuracy. Notably, simultaneous sampling of both $CD{F}_{d}^{dist}$ and $CD{F}_{d}^{pop}$ reduced model accuracy compared to sampling from either distribution independently. These results suggest that comprehensive sampling of all contributing CDFs is required to achieve accurate and robust modeling.

The models in this study were calculated using histological data derived from a single metastatic colorectal tumor treated with 3.2 GBq (60 million microspheres) of SIR-spheres. Nonetheless, modeling may be generalized given the agreement in ${C}_{dia}$, ${C}_{dist}$, and ${C}_{pop}$ across liver cancer pathologies and microsphere products [9, 23]. For example, Pillai showed when doubling the microsphere concentration, ${C}_{pop}$ was stable for 27 μm diameter polystyrene microspheres in hepatic VX2 tumor models [24]. Pasciak showed the median of ${C}_{pop}$ was largely independent of microsphere concentration in a porcine healthy liver model [15]. Hoberg found ${C}_{pop}$ was 3 microspheres/cluster for two patients having intrahepatic cholangiocarcinoma treated with SIR-spheres [25]. Kennedy discovered that SIR-spheres and TheraSphere microspheres formed clusters generally having between 1 and 4 microspheres each [9]. In Pasciak’s porcine model, ${C}_{dia}$ for TheraSphere microspheres agreed with SIR-spheres [15]. It was also shown in that study that when the microsphere concentration was increased, ${C}_{dia}$ and ${C}_{pop}$ remained relatively constant while ${C}_{dist}$ was reduced [15]. These results suggest the methodology presented is in this work is largely independent of microsphere product and liver cancer pathology, and could be implemented to generate models given user-specified pathological data. These new models could be employed in future studies on a patient-specific basis to improve treatment planning for a given liver cancer pathology and microsphere product (e.g., hepatocellular carcinoma treated with TheraSphere microspheres).

Seven unique models were examined in this study to investigate the impact of the microsphere deposition algorithm complexity on the accuracy of absorbed dose metrics. While the uniform microsphere distribution may be intuitive and easy to visualize, it proves inaccurate and suggests D_mean_ may not be an appropriate metric at the microscopic scale. By examining dose metric accuracy, this study also demonstrated that each microsphere cluster parameter is required for accurate modelling. However, some parameters were more relevant in terms of dose metric accuracy and are responsible for modeling different aspects of the dose distribution (${C}_{pop}$ for dose hot spots, e.g.). Understanding the relative importance of ${C}_{dia}$, ${C}_{dist}$, and ${C}_{pop}$ may assist in identifying relevant microsphere cluster parameters for computational modeling of fluid dynamics [26, 27]. The impact of parameter reduction was also shown to influence the computational burden. However, the most accurate models were associated with the longest computation times.

The accuracy of the microscopic dose distribution not only depends on how well the modeled microsphere distribution represents actual *in vivo* microsphere distributions, but also on the accuracy of the ^90^Y DVK required to calculate the absorbed dose [28]. As shown in figure 2(b), there is excellent agreement between the TOPAS-based DVK and the ICRU reference DPK. Near the source voxel, discrepancies are expected from differences in the dose scoring geometry between DVKs and DPKs (voxels versus spherical shells, respectively). Beyond the source voxel, there is agreement between the profiles up to the maximum range of a ^90^Y beta particle where there are fewer interactions. This is inconsequential, however, since the absorbed dose to these voxels are roughly five orders of magnitude less than the dose in central voxel. Due to the high-resolution modeling in this study where the voxel size is equal to the average diameter of a ^90^Y microsphere, the source voxel of the DVK should have a material composition specific to the commercial microsphere for accurate dosimetry.

**Figure 6.**
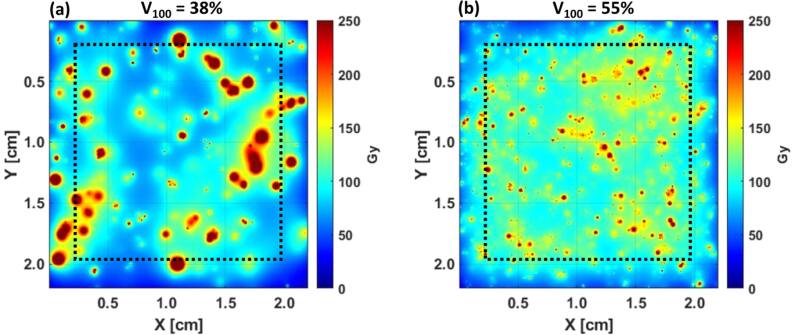
Dose distributions in ${M}_{ref}$ with varying microsphere specific activity and microsphere concentration having the same mean absorbed dose of 100 Gy. Dose metrics were calculated for the volume within the dashed box, representing the region capable of achieving charged particle equilibrium. (a) Microsphere concentration of 5 × 10^3^ microspheres ml^−1^ and specific activity of 500 Bq/MS. (b) Microsphere concentration of 50 × 10^3^ microspheres/mL and specific activity of 50 Bq/MS.

The motivation for this study was to gain insight into the microscopic characteristics of the ^90^Y dose distribution and build a robust framework to further understand the relationship between microdosimetry and macrodosimetry-based clinical outcomes. To this end, the utility of this work can be demonstrated by considering two microsphere distributions from ${M}_{ref\,}$ having a D_mean_ of approximately 100 Gy: (1) microspheres have a specific activity of 500 Bq/MS and a concentration of 5,000 microspheres/mL and (2) microspheres have a specific activity of 50 Bq/MS and a concentration of 50,000 microspheres/mL. A slice through both dose distributions is shown in figure 6. Dose metrics were calculated for the volume within the dashed box, representing the region capable of achieving charged particle equilibrium. Despite having an equivalent D_mean_, the dose distribution uniformity is greater in the case having a 10x larger microsphere concentration. While the first case produces dose hot spots throughout the volume, the dose to these hot spots (1000’s of Gy) is far greater than the dose required destroy tumor cells (100’s of Gy) [1–3]. Quantitatively, the percent volume receiving at least 100 Gy (V_100_) is equal to 38% in the first case and 55% in the second case, suggesting the second case is likely to produce an improved tumor response. Clinical data are required to verify this claim.

Model accuracy was limited by the underlying assumptions of the histological data. It was assumed that there was no correlation between cluster size and number of microspheres per cluster when previous research found that the measures for cluster population, cluster size, and distance between clusters were not strictly independent [7]. Rather, there was a tendency for clusters with larger populations to have a larger size and to be closer other clusters [15]. Another limitation in developing models for microdosimetry is a lack of explant data to refine the PDFs describing ${C}_{dia}$, ${C}_{dist}$, and ${C}_{pop}$ in normal liver tissue and in tumors from more cancer types (e.g. HCC). A final limitation was imposed by the manual extraction of the histological data from the original research paper published in 2000 [7]. This process was time consuming and introduced a small degree of uncertainty into the PDFs. This justifies the need for analytic functions to describe the PDFs. The determination of analytic functions, as well as the availability of the high-resolution ^90^Y DVK, should ease model implementation for future investigators.

Pasciak’s computational study investigated the effect of microsphere distribution at the mm-scale on the tumor dose distribution [14]. Although Pasciak’s stochastic algorithm was used, there are several salient aspects to this study. First, this study challenged that assumption of needing to individually sample PDFs describing ${C}_{dia}$, ${C}_{dist}$, and ${C}_{pop}$ by reporting that sampling ${C}_{dist}$​ is critical for accurately modeling low-dose regions, sampling ${C}_{pop}$ is critical for resolving absorbed dose hot spots, and sampling from ${C}_{dia}$​ had minimal impact on model accuracy. Our study also provided a comprehensive picture of the model’s accuracy and computational cost. Second, our study developed continuous PDFs and validated their use, significantly enhancing the reproducibility and applicability of stochastic modeling for future investigations. Compared to Pasciak’s study that used 100 μm voxels, this work utilized 30 μm voxels with > 3x improved spatial resolution that allows for a single microsphere to occupy a voxel, enabling more accurate and precise microdosimetry. Overall, these improvements allow for a more accurate evaluation of how microscopic parameters (microsphere concentration, specific activity) impact the microscopic dose distribution. In terms of clinical impact, these improvements provide a means to further understand the relationship between microdosimetry and macrodosimetry-based clinical outcomes.

Subsequent modeling studies will utilize ${M}_{ref}$ to investigate the impact of clinically relevant combinations of microsphere concentration and specific activity on patient outcome. Specific dose-volume metrics will be compared through statistical testing to isolate combinations capable of achieving pathological necrosis in tumors and minimizing normal tissue toxicity, given an absorbed dose threshold. Ultimately, microdosimetry modeling in ^90^Y-RE could provide the means to establish a model-based dose response relationship.

## 5. Conclusion

In this study, a stochastic microsphere deposition algorithm was implemented based on sampling of histological data to generate various *in vivo* microsphere distribution models. Continuous analytical functions were chosen to describe the discrete probability distributions functions for microsphere cluster diameter (${C}_{dia}$), distance between clusters (${C}_{dist}$), and cluster population (${C}_{pop}$) to facilitate ^90^Y-radioembolization microdosimetry modeling and reproducibility. A high-resolution (30 μm pitch) ^90^Y dose-voxel kernel was generated and validated for enabling microdosimetry calculations. The impact of microsphere deposition algorithm complexity for modeling *in vivo* microsphere depositions and microdosimetry was evaluated. Sampling the ${C}_{dist}$ was essential for accurate modeling of low dose regions, sampling ${C}_{pop}$ was essential for accurate modeling of absorbed dose hot spots, and sampling ${C}_{dia}$ had a minimal impact on model accuracy. However, with combined sampling of ${C}_{dist}$ and ${C}_{pop}$, the resulting model was less accurate than models sampling either ${C}_{dist}$ or ${C}_{pop}$ alone—suggesting that all microsphere cluster parameter should be sampled for accurate modeling.

## Data Availability

All data that support the findings of this study are included within the article (and any supplementary files).
